# Frailty Detection in Older Adults with Diabetes: A Scoping Review of Assessment Tools and Their Link to Key Clinical Outcomes

**DOI:** 10.3390/jcm13175325

**Published:** 2024-09-09

**Authors:** Ernesto Guevara, Andreu Simó-Servat, Verónica Perea, Carmen Quirós, Carlos Puig-Jové, Francesc Formiga, María-José Barahona

**Affiliations:** 1 Department of Geriatrics, Hospital Universitari Mútua-Terrassa, University of Barcelona, 08007 Barcelona, Spain; 2 Department of Endocrinology, Hospital Universitari Mútua-Terrassa, University of Barcelona, 08007 Barcelona, Spain; asimo@mutuaterrassa.cat (A.S.-S.); vperea@mutuaterrassa.cat (V.P.); cquiros@mutuaterrassa.cat (C.Q.); cpuig@mutuaterrassa.cat (C.P.-J.); 3 Department of Internal Medicine, Instituto de Investigación Biomédica de Bellvitge (IDIBELL), Hospital Universitari de Bellvitge, University of Barcelona, 08007 Barcelona, Spain

**Keywords:** frailty, older-adults, elderly, diabetes, assessment-tools, hyperglycemia, hypoglycemia, metabolic-phenotypes

## Abstract

**Objectives:** With the increasing prevalence of diabetes and frailty among older adults, there is an urgent need for precision medicine that incorporates comprehensive geriatric assessments, including frailty detection. This scoping review aims to map and synthesize the available evidence on validated tools for detecting pre-frailty and frailty in community-dwelling elderly individuals with diabetes and outpatient diabetes patients. Specifically, it addresses: (1) What validated tools are available for detecting pre-frailty and frailty in this population? (2) How are these tools associated with outcomes such as glycemic control, hypoglycemia, and metabolic phenotypes? (3) What gaps exist in the literature regarding these tools? **Methods:** The review followed PRISMA-ScR guidelines, conducting a systematic search across PubMed, Cochrane Library, and Web of Science. The inclusion criteria focused on studies involving individuals aged 70 years and older with diabetes, emphasizing tools with predictive capacity for disability and mortality. **Results:** Eight instruments met the inclusion criteria, including the Frailty Index, Physical Frailty Phenotype, and Clinical Frailty Scale. These tools varied in domains such as physical, psychological, and social aspects of frailty and their association with glycemic control, hypoglycemia, and metabolic phenotypes. The review identified significant gaps in predicting diabetes-related complications and their clinical application. **Conclusions:** Routine management of older adults with diabetes should incorporate frailty detection, as it is crucial for their overall health. Although widely used, the reviewed tools require refinement to address the unique characteristics of this population. Developing tailored instruments will enhance precision medicine, leading to more effective, individualized interventions for elderly individuals with diabetes.

## 1. Introduction

Diabetes is a pressing global health issue, affecting over 693 million people worldwide in 2017, with a global prevalence of 8.4% [1]. In Spain, the prevalence is 13.8% among individuals over 18 years of age [2], increasing significantly with age, especially among those over 75 years old [3]. This prevalence is projected to rise, particularly among older adults, driven by factors such as increased life expectancy, aging populations, and suboptimal health habits [4].

The burden of diabetes largely stems from its complications, both macrovascular (e.g., heart attacks and strokes) and microvascular (e.g., blindness, lower limb amputation, and renal failure) [1,5]. Recent advancements in diabetes management, including the development of new medications and improved monitoring technologies, have enhanced treatment outcomes [6,7,8,9,10]. Despite these advancements, their implementation into clinical practice often lags, particularly for older adults [11,12].

Older adults with diabetes represent a unique and heterogeneous group [13], often dealing with comorbidities, polypharmacy, and complications that predispose them to conditions such as frailty [3,14,15].

Frailty, characterized by generalized functional decline [16], is a key concept in healthcare, especially for aging populations. It is defined as a multidimensional clinical syndrome characterized by reduced physiological and functional reserves, diminished adaptive capacities, and heightened vulnerability to low-intensity stressors, which can be potentially reversed or attenuated through individualized interventions [17,18]. Frailty serves as an indicator of biological age, distinct from chronological age, and should not be equated with disability [19]. It exists on a continuum, ranging from physical frailty to functional dependency, and it is influenced by the presence of comorbidities [20,21].

Frailty is a syndrome that involves multiple dimensions, including physical, biological, psychological, social, and economic factors [22]. It encompasses five essential components: vulnerability, genesis, characteristics, phenotype, and adverse health-related outcomes, which collectively define the condition [23]. The diversity in definitions highlights the absence of a universally accepted definition, complicating its consistent application in research and practice [22]. Importantly, frailty is not an inevitable consequence of aging but arises from the interaction of biological, psychological, and social factors, making it a complex condition [23]. Factors such as age, comorbidities, nutritional deficits, and sarcopenia are commonly associated with frailty, emphasizing the importance of a multidimensional approach to its definition and management. This syndrome is associated with increased risks of disability, hospitalization, and premature mortality, further underscoring the need for a unified definition that integrates physical, social, and psychological components for effective assessment and management in clinical settings [22,23].

The development of frailty involves several physiological systems, including the metabolic, neuroendocrine, and cardiovascular systems [24,25,26,27,28,29,30,31,32,33,34,35,36,37,38,39,40,41]. In older adults with diabetes, frailty is associated with adverse outcomes such as disability, hospitalizations, and increased mortality [42,43]. Identifying frailty in this population is crucial for guiding individualized treatment goals for glycemic control, lipid management, body weight control, and blood pressure control [13], but also for addressing the broader bio-psycho-social dimensions of frailty [23]. Frailty encompasses a complex interplay of physical, psychological, and social factors, which together influence the overall health and quality of life in diabetic patients. This comprehensive approach, which includes improving therapeutic adherence and addressing psychological and social well-being, is essential for developing personalized interventions that enhance both clinical outcomes and quality of life for these patients [22].

In Spanish older adults, prevalence rates of frailty range from 8.4% to 34.7% [44,45,46,47,48]. Among older individuals with diabetes, frailty prevalence is particularly high [49], as observed in the Beijing Longitudinal Study of Aging II, which found that 19.3% of older adults with diabetes were frail compared to 11.9% without diabetes [5].

Given the growing prevalence of diabetes and frailty among the older adult population, there is an urgent need for precision medicine that incorporates comprehensive and dynamic geriatric assessments, including frailty detection.

This scoping review aims to map and synthesize the available evidence on validated tools for the detection of pre-frailty and frailty in community-dwelling elderly individuals living with diabetes, as well as outpatient diabetes patients. Specifically, the review addresses three key questions: (1) What validated tools are currently available for the detection of pre-frailty and frailty in this population? (2) How are these tools associated with critical outcomes such as glycemic control, hypoglycemia, and metabolic phenotypes? (3) What gaps exist in the literature regarding the use of these tools?

By exploring these questions, the review seeks to identify areas where further research is needed to enhance precision medicine in managing diabetes and frailty in this population.

## 2. Methods

### 2.1. Protocol and Registration

This scoping review was conducted in accordance with the Preferred Reporting Items for Systematic Reviews and Meta-Analysis for Scoping Reviews (PRISMA-ScR) statement [50], following the methodological framework established by Arksey and O’Malley [51,52,53]. Their framework provides a systematic approach for scoping studies, which involves identifying, selecting, and analyzing relevant literature to map the existing evidence on a particular topic. By adhering to this framework, our review aims to offer a comprehensive overview of the available tools for frailty detection in older adults with diabetes, ensuring methodological robustness and transparency. While we followed the PRISMA-ScR guidelines to ensure transparency and rigor in our reporting, it is important to note that this scoping review was not registered in a publicly accessible protocol database, as protocol registration is not mandatory for scoping reviews.

### 2.2. Eligibility Criteria

The target population for this scoping review included community-dwelling individuals and outpatients, aged 70 years and older, living with diabetes (type 1, type 2, or Latent Autoimmune Diabetes of Adults), regardless of gender and ethnicity.

We included studies that evaluated both physical frailty and frailty due to the accumulation of deficits. We considered scales or indices that clearly identified the state of pre-frailty, as well as tools or instruments with predictive capacity for disability and mortality.

However, we excluded studies that used adaptations of the original Physical Frailty Phenotype operationalized by Fried [20], the Rockwood’s Frailty Index [21], the Clinical Frailty Scale [54], or the Frailty Trait Scale [55]. Additionally, we did not include instruments for frailty detection via self-report or those validated exclusively in hospital settings, nursing homes, or long-term care facilities. Instruments used exclusively for preoperative evaluation or in emergency care settings were also excluded.

### 2.3. Information Sources

We conducted a comprehensive literature search, rigorously assessing relevant articles by querying three major electronic databases: PubMed, Cochrane Library, and Web of Science. The primary objective was to identify peer-reviewed articles published between January 2000 and December 2023. The final search was completed on January 15, 2024. In addition, a secondary search was performed to review the bibliographic references cited in the included studies. To further enhance the thoroughness of the literature review, we conducted a manual examination of citations within the retrieved articles.

### 2.4. Search

A preliminary search suggests that existing studies on predictive models of complications associated with diabetes primarily focus on macrovascular complications, with limited attention given to other diabetes-related complications, such as geriatric syndromes. These initial findings informed the development of our search strategy and the establishment of our inclusion criteria. We conducted a comprehensive search using both free text and Medical Subject Headings (MeSH) for various forms of the following terms (in titles and abstracts): (“Aged” OR “Elderly” OR “Older”) AND (“Frailty”) AND (“Diabetes Mellitus”) AND (“Diagnosis” OR “Prevalence” OR “Outcome assessment”). Then we combined these items using AND with (“Metabolism” OR “Metabolic phenotype” OR “Insulin resistance” OR “Obesity” OR “Sarcopenia” OR “Lipids”) AND (“Hyperglycemia”) AND (“Hypoglycemia”) AND (“Disability evaluation” OR “Prognosis”) AND (“Mortality” OR “Death” OR “Survival rate”) AND (“Independent living” OR “Outpatients” OR “Ambulatory care”). The terms and truncated variants of the terms were then combined for study retrieval.

### 2.5. Selection of Sources of Evidence

Screening, the selection of articles, data extraction, and quality assessment were performed independently by two authors (E.G. and M.J.-B.), who analyzed the titles, abstracts, and full texts of the eligible studies. Discrepancies between the two reviewers were resolved jointly with a third author (F.F.).

To assess the quality of the included studies, we utilized the Joanna Briggs Institute (JBI) Global Checklist for Systematic Reviews and Research Syntheses, focusing on key aspects such as study design, bias, and the validity of the findings. This allowed us to ensure that the studies included were methodologically sound and provided reliable evidence.

In addition, we employed the Consensus-based Standards for the selection of Health Measurement Instruments (COSMIN) checklist to critically evaluate the measurement properties of the frailty assessment tools identified in our review. Specifically, we assessed the reliability, validity, and responsiveness of each instrument, which are essential criteria for determining their suitability and robustness in clinical settings. The COSMIN tool provided a structured approach to ensure that only high-quality instruments were included in our synthesis. We acknowledge that a limitation of this search strategy is the potential omission of contributions from non-English language scientific resources and journals.

**Figure 1:**
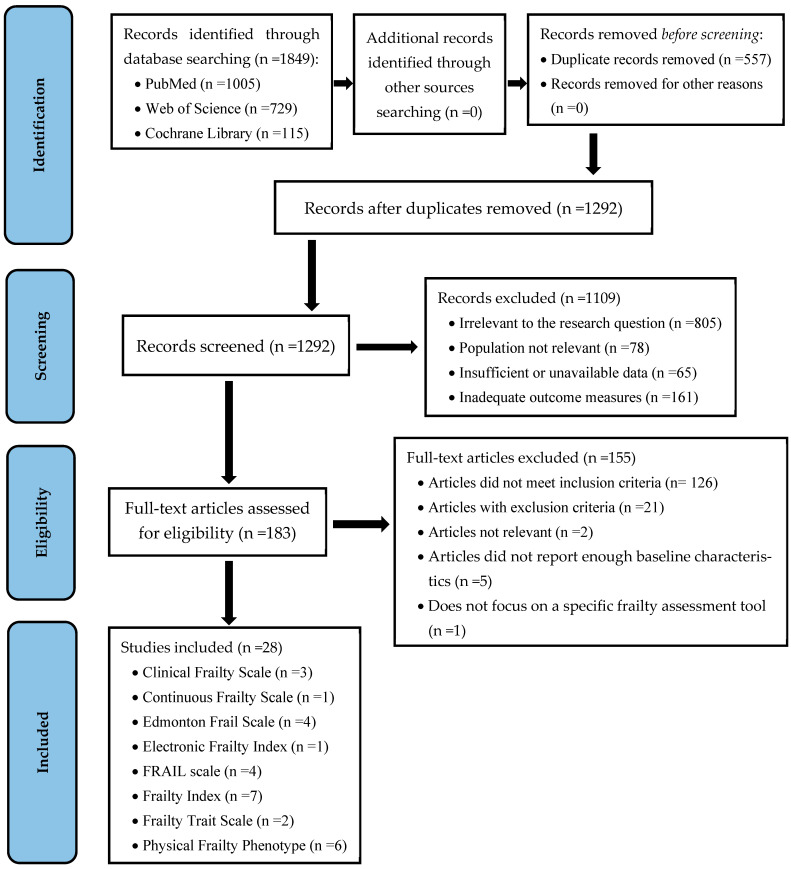
PRISMA flow diagram.

The selection process is summarized in the PRISMA flow diagram (Figure 1).

### 2.6. Data Charting Process

Data extraction was performed using a standardized charting form specifically designed for this review. The form was pilot-tested on a sample of five studies to ensure that it captured all relevant information, including study characteristics (e.g., year of publication, country of origin), participant demographics (e.g., age, sex), the frailty assessment tools used, and reported clinical outcomes related to glycemic control, hypoglycemia, and metabolic phenotypes. Two reviewers (E.G. and M.J.-B.) independently extracted data in duplicate, and discrepancies were resolved through consensus or by consulting a third reviewer (F.F.). The data charting process was iterative, with the form being updated as new variables emerged during the review.

### 2.7. Data Items

The specific data items extracted from each included study were as follows:

- Study characteristics: year of publication, country of origin, study design.
- Participant demographics: age, sex, sample size.
- Frailty assessment tools: type of tool, operationalization, context of use.
- Clinical outcomes: associations with glycemic control (e.g., HbA_1c_ levels), incidence of hypoglycemia, and characterization of metabolic phenotypes. All extracted data were documented meticulously to ensure the reliability and accuracy of the review’s findings.

### 2.8. Synthesis of Results

The results were synthesized narratively, focusing on the range of frailty assessment tools used in older adults living with diabetes and their reported associations with key clinical outcomes. Due to the heterogeneity of the included studies, a meta-analysis was not feasible. Instead, the synthesis aimed to provide a comprehensive overview of the existing evidence, highlighting trends and gaps in the current literature.

## 3. Results

In our search for instruments to detect frailty syndrome in community-dwelling individuals with diabetes as well as outpatients with diabetes aged 70 years and older, regardless of gender and ethnicity, we relied on systematic reviews by Faller et al. [56], Rasiah et al. [57], Aguayo et al. [58], de Vries et al. [59], and Sternberg et al. [60]. These reviews described 35, 32, 51, 20, and 22 instruments, respectively, as well as 67 instruments reviewed by Buta et al. [61].

The instruments assessed were highly heterogeneous and differed primarily in terms of their construction and/or validation for use in different settings, including emergency departments, hospitalized patients, outpatient settings, community-dwelling elderly people, individuals in long-term care social-health centers, and institutionalized older adults in nursing homes. These instruments assess different domains of frailty (physical, psychological, and social), and not all of them detect pre-frailty, nor predict disability and mortality.

Therefore, we conducted a scoping literature review to identify validated screening tools for pre-frailty and frailty used in older adults with diabetes residing in the community (or as outpatients), focusing on tools with predictive capacity for disability and mortality. We identified eight instruments that met our inclusion criteria, including the Clinical Frailty Scale (CFS) [54], Continuous Frailty Scale [62], Edmonton Frail Scale (EFS) [63], Electronic Frailty Index (eFI) [64], FRAIL Scale [65], Physical Frailty Phenotype (PFP) [20], Frailty Index (FI) [21,66] and Frailty Trait Scale (FTS) [55]; see Table 1.

In our research, we identified six instruments that also detected both pre-frailty and frailty: the Frailty Screening Questionnaire [84], the Geriatric Functional Evaluation [85], the SHARE Frailty Instrument [86], the SHARE Frailty Instrument 75+ [87], the Short Physical Performance Battery [88], and the Study of Osteoporotic Fractures criteria [89]. These instruments were validated and used in older adults residing in the community and demonstrated predictive capacity for disability and mortality. However, to our knowledge, they have not been studied in people with diabetes.

The most commonly used and cited frailty screening instruments in studies involving people with diabetes are: the Clinical Frailty Scale, the Frailty Index, Physical Frailty Phenotype, the FRAIL Scale, and the Edmonton Frail Scale [90,91,92,93].

The FRAIL scale has not been implemented in studies of glycemic control, but it has been associated with metabolic phenotypes [94] and hypoglycemia [74]. Conversely, the Edmonton Frail Scale has not been studied in relation to hypoglycemia, but it has been correlated with metabolic phenotypes [94] and glycemic control [71,72].

The Continuous Frailty Scale [62], the Electronic Frailty Index [64], and the Frailty Trait Scale [55,80] exhibit high sensitivity for detecting pre-frailty and frailty and are valid predictors of disability and mortality in people with diabetes. However, these instruments have not been used in assessing glycemic control, hypoglycemia, or metabolic phenotypes.

### 3.1. Frailty and Glycemic Control

P. Hanlon et al., in a frailty analysis of 20,566 people with diabetes, revealed that the risk of all-cause mortality among individuals identified as “frail” using Fried’s Physical Frailty Phenotype was higher in those with higher initial levels of HbA_1c_ [13]. This finding suggests a “J-shaped” mortality curve when frailty and HbA_1c_ are associated.

Several studies have used the PFP to explore the association between poorly controlled glycemia defined as “elevated glucose levels” and measured with HbA_1c_ [81]. One of the earliest studies by Blaum et al. [29] suggested a link between elevated glucose levels and frailty, emphasizing the importance of maintaining good glycemic control to prevent frailty. The study found that HbA_1c_ levels in the range of 6.5% to 6.9% were consistently associated with an increased risk of frailty. This association remained significant for HbA_1c_ levels up to 8.9%, with a markedly stronger risk observed at levels of 9.0% and above.

These findings underscore the need for careful glycemic management in older adults to reduce the risk of developing frailty.

Bilgin et al. found that fasting plasma glucose (*p* = 0.02) and HbA_1c_ (*p* = 0.04) were significantly higher in the frail patient group compared to patients without frailty, as measured by the Edmonton Frail Scale [72].

In a study by Atif et al. [68], the mean HbA_1c_ level among all participants was reported as 7.8 ± 1.5%. Glycemic control was particularly suboptimal among participants with frailty, as assessed by the Clinical Frailty Scale. Notably, nearly two-thirds (64.2%) of patients aged ≥62 years had elevated HbA_1c_ levels, a significant finding given that life expectancy in Pakistan is approximately 66 years. Additionally, 67.4% of patients with comorbidities and 73.5% of those with diabetes complications had HbA_1c_ levels exceeding the target range. The study also highlighted that over 70% of patients with severe depression, mild cognitive impairment, low physical function, malnutrition, and severe pain failed to achieve optimal HbA_1c_ levels. These findings illustrate the considerable challenges in managing glycemic control among older adults with frailty, underscoring the necessity for personalized interventions in this population.

This study emphasizes the complex interplay of factors that contribute to frailty, extending beyond just the biological to include psychological, social dimensions, and treatment adherence. Frailty, therefore, must be understood as a condition that arises from various intersecting factors rather than an inevitable consequence of aging. Thus, a holistic approach that integrates these multiple dimensions is essential for developing effective interventions tailored to the needs of older adults with diabetes.

Using the CFS, MacKenzie et al. [69] found that individuals with diabetes and moderate to severe frailty had lower glucose levels than those with no frailty or mild frailty, as assessed by serum glucose levels at the time of hospitalization. However, HbA_1c_ levels did not significantly vary in relation to the degree of frailty in diabetic patients.

Conversely, Yanagita et al. investigated the association between HbA_1c_ levels and frailty, also using the CFS, in older Japanese individuals with T2D. Their analysis revealed that lower HbA_1c_ levels were significantly associated with greater frailty severity, as evidenced by an inverse correlation between HbA_1c_ and CFS scores (r = −0.31, *p* < 0.01). This finding suggests that poor glycemic control characterized by low HbA_1c_ may be linked to an increased risk of frailty in this population [67].

Zaslavsky et al. observed a U-shaped relationship in older adults with diabetes, indicating that both lower and higher glucose levels were associated with an increased risk of frailty [82]. This relationship was measured using Fried’s PFP model. In this studied population, glucose levels below 160 mg/dL and above 180 mg/dL were associated with a higher risk of frailty, with the lowest risks observed at glucose levels of approximately 170 mg/dL.

Similarly, in an analysis of the English Longitudinal Study of Ageing, Aguayo et al. found that higher levels of HbA_1c_ were prospectively associated with increased frailty status (consistently measured using the FI, the EFS, and the PFP). Specifically, there was a significant positive association between higher HbA_1c_ levels (β = 4.2 [95% CI 2.5, 5.9]) and elevated frailty scores, indicating that poor glycemic control may exacerbate frailty over time [71]. Conversely, the study observed that among individuals with diabetes at younger ages, lower HbA_1c_ levels exhibited a trend toward association with higher levels of frailty, suggesting a complex relationship between glycemic control and frailty. Notably, the HbA_1c_-age interaction was significant and positive, implying that the association between HbA_1c_ levels and frailty tended to strengthen with age.

In an exploratory analysis by Simpson et al., involving 4169 participants from the Action for Health in Diabetes (Look AHEAD) trial over an 8-year follow-up period, lower HbA_1c_ levels among individuals with diabetes were associated with slower biological aging. This was reflected through deficit accumulation measured using the Frailty Index [78].

Additionally, a systematic review with meta-analysis by Crabtree et al. found that intensive glycemic control in frail older adults with T2D did not significantly impact all-cause mortality but was associated with a higher risk of hypoglycemia [95].

### 3.2. Frailty and Hypoglycemia

Nguyen et al., in a post-hoc analysis of the Action in Diabetes and Vascular Disease: Preterax and Diamicron Modified Release Controlled Evaluation (ADVANCE) trial, observed that frailty status in individuals with T2D predisposes them to severe hypoglycemia, regardless of whether glycemic control is intensive or not, and regardless of age [77].

In this analysis, the unadjusted hazard ratio (HR) of intensive glycemic control for severe hypoglycemia was 1.86 (95% CI: 1.42–2.44). This impact was greater in frail participants (HR 2.18, 95% CI: 1.37–3.48) compared to non-frail participants (HR 1.73, 95% CI: 1.24–2.40); although this result was not statistically significant (*p* = 0.419). This finding highlights that frailty, rather than chronological age, may be a determining factor in susceptibility to severe hypoglycemia in individuals with T2D, irrespective of the intensity of glycemic control.

In a study by Chao et al. [75], among individuals with diabetes who experienced episodes of hypoglycemia prior to inclusion, it was observed after an average follow-up of 2.68 years that 1.6% developed incident frailty, as measured using a variant of the FRAIL scale. Additionally, mortality in the hypoglycemia group was twice as high compared to the group without hypoglycemia. However, patients who experienced hypoglycemia prior to inclusion had a higher prevalence of chronic kidney disease, cardiovascular diseases including heart failure, cerebrovascular disease, parkinsonism, and gout than those who did not experience hypoglycemia. Notably, 28.8% of patients who experienced previous hypoglycemia were taking antipsychotics at the time of randomization, although the prevalence of cognitive impairment or dementia was not specified. Importantly, in this study, episodes of hypoglycemia were not recorded in either group during follow-up.

### 3.3. Frailty and Metabolic Phenotypes

Regarding body weight, Hyde et al. found a U-shaped association between frailty and body mass index (BMI) when using the Frailty Index as a continuous measure [76]. Both underweight and people with obesity had higher Frailty Index scores compared to those with normal body weight.

In a study by García-Esquinas et al. involving older individuals from the Seniors-ENRICA cohort in Spain, an increased risk of frailty was observed in people with diabetes after 3.5 years of follow-up. This study linked the Physical Frailty Phenotype with obesity, hypertriglyceridemia, and poor glycemic control as key predisposing factors for frailty [81].

On the other hand, Akan et al. found that frail individuals, as identified by the FRAIL scale, had higher BMIs compared to non-frail patients. Additionally, those identified as frail by the EFS had larger abdominal circumferences compared to their non-frail counterparts [73].

Metabolic Syndrome (MetS) is associated with frailty [96], as observed in the Aspirin in Reducing Events in the Elderly (ASPREE) study. In this study, MetS was associated with a 22% and 66% higher likelihood of being pre-frail and frail, respectively, at the study’s inception [79]. Interestingly, frailty may be a more accurate predictor of mortality than MetS in older individuals, as suggested by an analysis using the Frailty Index [97]. The ASPREE study also found that frailty, measured by the FI and the PFP, was associated with reduced rates of disability-free survival, including outcomes such as incident dementia, persistent physical disability, or death from any cause [79].

In a study by Yanagita et al. [67], older individuals with T2D and frailty, as detected by the CFS, had significantly lower average values of body weight, albumin, and HDL cholesterol compared to those without frailty. Although the impact of sarcopenia was not reported, it was noted that risk factors for MetS could shift from unfavorable to favorable factors in old age, a phenomenon termed reverse metabolism [36,67]. Frail patients in this study also had lower levels of hemoglobin and systolic blood pressure. Notably, among patients without frailty, with an average age of 75.2 ± 6.2 years, there was a higher percentage of individuals receiving statin therapy compared to the frailty group. The research also identified low body weight and low levels of albumin, HDL cholesterol, and total cholesterol as significant risk factors for frailty, as measured by the CFS. Additionally, it associates frailty with low HbA_1c_ levels, suggesting that malnutrition could play a key role in the onset of hypoglycemia and frailty. It is important to note that a high number of patients were taking sulfonylureas (33%).

In the study by Atif et al., it was observed that older adults with suboptimal control of T2D and associated frailty (measured with the CFS) had a higher prevalence of malnutrition and hypoglycemia, which led to a greater predisposition to falls [68].

In an exploratory analysis by Simpson et al., involving 4169 participants from the Action for Health in Diabetes (Look AHEAD) trial with an 8-year follow-up, it was found that weight loss among individuals with diabetes may slow down the aging process, as measured by the accumulation of deficits using the FI [78]. This suggests that intentional weight loss achieved through nutritional interventions, physical exercise, and even pharmacological approaches could be beneficial in reversing frailty and reducing cardiovascular-metabolic risk, provided it is not associated with malnutrition or sarcopenia.

Bilgin et al. did not find an association between frailty as measured by EFS and body weight, BMI, or waist circumference. However, they observed an inverse correlation between frailty and serum albumin levels (*p* = 0.002) and estimated Glomerular Filtration Rate by serum creatinine (*p* < 0.001) [72]. These associations could be related not to malnutrition but to low-grade chronic inflammation and oxidative stress, which are mediators of sarcopenia [94]. Additionally, hyperglycemia observed in the study patients could also be related to insulin resistance [98].

## 4. Discussion

Despite exhaustive reviews of frailty instruments in recent years, including efforts to align their elements with the International Classification of Functioning, Disability, and Health [49], a standard frailty assessment tool for older adults living with diabetes has yet to be established [17,99]. The International Conference on Frailty and Sarcopenia Research currently recommends the use of three scales for frailty identification in the general population: the Clinical Frailty Scale, the FRAIL scale, and the Edmonton Frail Scale [100]. However, it does not mention the use of any specific scale for people with diabetes, revealing the significant heterogeneity and lack of consensus in existing recommendations.

Based on our findings, the three most widely used screening instruments for identifying pre-frailty and frailty in elderly individuals with diabetes are the Frailty Index [13,15,71,76,77,78,79,80], the Physical Frailty Phenotype [13,71,79,81,82,83], and the Clinical Frailty Scale [67,68,69].

These tools have demonstrated predictive capacity for disability and mortality in studies involving older adults with diabetes and have also been used to associate frailty with glycemic control, hypoglycemia, and metabolic phenotypes, particularly in outpatient settings or among community-dwelling individuals.

The lack of consensus, possibly semantic [52], regarding an indisputable definition of frailty, coupled with a limited understanding of the different metabolic phenotypes that older adults with diabetes may present, undermines precision medicine [99]. While the primary goals of glycemic and lipid control, weight reduction, and cardio-renal protection remain paramount, it is essential to recognize that many individuals may require intensified pharmacological treatments alongside personalized nutrition and exercise programs.

Conversely, for individuals with high functional dependency and limited life expectancy, a more conservative approach to glycemic controls, simplified treatment regimens, avoiding polypharmacy, and focusing more on controlling geriatric syndromes may be more beneficial.

Frailty was not designed to “simply predict.” Its construct is based on the assumption that the traditional medical approach is insufficient to address the multiple, heterogeneous, complex, circumstantial, and individual needs of older adults. Consequently, selecting a frailty assessment tool should not be limited to “choosing one of the many validated instruments” but should actively aim towards “the validated tool that best aligns with individuals’ unique characteristics considering their pathologies and clinical context. This approach ensures that the chosen tool furnishes the most pertinent information for informed decision-making in the future” [101].

Considerable heterogeneity exists within populations identified as frail, highlighting the necessity of individualized decision-making for patients. This process should take into account the outcomes of the Comprehensive Geriatric Assessment and the specific measure used to assess frailty, rather than relying solely on “general recommendations for frailty” [13].

While the vast majority of existing literature has not shown clear benefits of the association between hyperglycemia and frailty, clinical practice guidelines recommend higher glycemic targets in people with frailty.

It is important to note that incorrectly interpreting falsely elevated HbA_1c_ values may lead to intensified pharmacological treatment, potentially resulting in frequent episodes of hypoglycemia and subsequent complications, ultimately worsening prognosis. Additionally, recurrent episodes of hypoglycemia, particularly in patients with high glycemic variability, may manifest as a low HbA_1c_. This occurs because HbA_1c_ fails to capture the daily fluctuations in blood glucose levels as effectively as continuous monitoring does [102].

Therefore, alongside HbA_1c_ monitoring, it is advisable to implement daily capillary glucose control. For patients at high risk of hypoglycemia, such as those on insulin therapy, Continuous Glucose Monitoring (CGM) is strongly recommended.

Longitudinal studies on the consequences of glycemic control in frail patients remain notably scarce. However, it is noteworthy that despite this scarcity, most guidelines emphasize the importance of individualizing glycemic control targets and recommend HbA_1c_ levels based on clinical characteristics, life prognosis, and functional autonomy of patients. Stricter HbA_1c_ levels are advised for functionally independent individuals, while more lenient goals are suggested for frail patients with functional dependencies for activities of daily living [103,104,105,106,107,108].

There remains uncertainty regarding whether strict glycemic control per se worsens the prognosis of older patients with diabetes and frailty, or if it is the hypoglycemia that occurs in this attempt. It is essential to understand that poor glycemic control characterized by elevated HbA_1c_ levels will worsen frailty through mechanisms such as sarcopenia, microvascular complications (particularly retinopathy and neuropathy), and a state of low-grade chronic inflammation [27]. However, what remains uncertain is the precise point in the evolution of diabetes with its complications and comorbidity, as well as the extent and duration of frailty, at which reversing poor glycemic control can effectively reverse frailty.

In our opinion, perhaps it is time to consider the prevailing message that “glycemic control should be lax in frail individuals due to the risk of hypoglycemia”. Instead, we propose that “in frail individuals glycemic control should be individualized, being somewhat strict in some cases while others may benefit from a more lenient approach, all while prioritizing the avoidance of hypoglycemia”. However, achieving this requires future clinical trials and the development of medication that promotes effective glycemic control without inducing hypoglycemia, which would provide the necessary insights.

Hypoglycemia serves as a surrogate marker for frailty [109], as its principal consequences, such as falls [102] and cognitive impairment [110], can lead to physical and cognitive frailty [13], respectively. The deleterious effects of hypoglycemia in older individuals are well-documented [111], contributing to an increased risk of cardiovascular events [112], cardiac arrhythmias, ischemic stroke, delirium [113], hospitalization [114], and mortality [112,115], with significant economic and social impact [116]. However, few studies have evaluated the correlation between frailty and hypoglycemia [90], with most focusing on the association between hypoglycemia and falls resulting in fractures [102].

The association between frailty and hypoglycemia remains controversial [75] within the scientific community and lacks robust empirical support to validate its veracity. The axiom linking them is mainly based on findings from clinical trials such as the Action to Control Cardiovascular Risk in Diabetes (ACCORD) trial, where participants aged over 80 were included, showing already prevalent high rates of hypoglycemia regardless of whether they were assigned to intensive or standard intervention arms. Across both intervention groups, the relative risk of severe hypoglycemia appeared consistent between younger and older participants. However, older participants exhibited higher absolute rates of severe hypoglycemia compared to their younger counterparts, irrespective of treatment intensity. It is worth noting that while intensive glucose reduction in the ACCORD trial was associated with increased cardiovascular risk and overall mortality in younger participants (those under 65 years old), its impact on older participants was more nuanced, showing a neutral effect [117].

The few studies examining the relationship between hypoglycemia and frailty have several limitations. These include the lack of prospective cohort studies that exclude other frailty confounders, such as malnutrition, sarcopenia, and chronic low-grade inflammation from comorbidities. Additionally, these studies often involve the use of hypoglycemia-inducing medications, such as sulfonylureas [74], which fail to detect pre-frailty at the time of inclusion, thus significantly increasing the incidence of frailty, the lack of HbA_1c_ values for all patients at inclusion, and do not utilize CGM, overlooking non-perceived hypoglycemia. Furthermore, the results are often derived from studies on specific populations (such as Asians), limiting the generalizability to other ethnicities [102].

Hypoglycemic events should always be avoided in older individuals with diabetes; however, hypoglycemia does not equate to frailty [90]. Without detecting frailty using validated scales, lax glycemic control should not be assumed to lead to frailty. It is worth noting that evidence from programs such as the Active Steps for Diabetes suggests that diabetes education can be effective in reducing HbA_1c_ and frailty [118]. Some older individuals with diabetes may benefit more from good glycemic control to reverse early-onset frailty [3,13,17,29,70,71,72,77,78,95]. The key question we pose is: What is the optimal tool for identifying and differentiating these older individuals with diabetes?

The available data on the relationship between aging, adiposity, sarcopenia, chronic inflammation, and other comorbidities with diabetes in older adults are scant and often contradictory. Further studies are essential to establish clear metabolic phenotypes and understand how these factors influence the pathophysiology of diabetes in this population.

This scoping review has some limitations. The heterogeneity of the assessment tools and their limited applicability in assessing glycemic control, hypoglycemia, or metabolic phenotypes hinder comparability. Additionally, reliance on clinical assessments, exclusion of non-English studies, and the use of cross-sectional designs limit both generalizability and causal inference. Furthermore, the lack of standardized definitions for frailty, inconsistent hypoglycemia data, along with the presence of potential confounders such as malnutrition, sarcopenia, chronic inflammation, and medication use, further complicate the interpretation of findings. These limitations underscore the urgent need for more robust, longitudinal research into the interplay between frailty and diabetes-related outcomes.

### 4.1. Clinical Implications

The high prevalence and clinical importance of frailty in older adults with diabetes are indisputable [90]. While frailty is not an inevitable consequence of aging and can potentially be prevented and reversed, making therapeutic decisions for frail older patients is extremely challenging. It should not only hinge on chronological age and functional status but on a myriad of factors, including comorbidities, polypharmacy, geriatric syndromes, psychological factors such as the degree of cognitive impairment, behavioral disorders associated with dementia, delirium, depression, and social factors such as social isolation and institutionalization. Incorporating considerations such as life expectancy, perceived quality of life, and personal preferences is vital in adopting a shared decision-making approach. This approach ensures that interventions are tailored to the individual needs and preferences. A comprehensive strategy should encompass physical exercise programs, nutritional recommendations, emotional support, and appropriate medication prescriptions [119].

Therefore, it is necessary to advance our understanding of how frailty should be defined and detected in older adults with diabetes for several reasons. Firstly, by targeting frailty, we aim to reverse or delay its progression, thereby mitigating physical disability and functional dependence. Ultimately, this approach seeks to enhance the health-related quality of life for older individuals with diabetes.

This scoping review highlights the importance of frailty as a robust predictor of disability-free survival, functional dependence, and mortality in older adults with diabetes. Frailty surpasses independent variables such as chronological age, comorbidity, or MetS [79]. Hence, integrating frailty status into the Comprehensive Geriatric Assessment of older adults with diabetes is not only beneficial but also essential. Additionally, recognizing frailty’s influence on treatment response [120] and the incidence of adverse events, such as severe hypoglycemia [77], underscores its importance in clinical trial design involving older individuals. Frailty serves as a marker of biological age, offering high sensitivity and predictive capacity for outcomes such as functional dependence, hospitalization, institutionalization, and mortality.

### 4.2. Future Perspectives

Glycemic targets and pharmacological therapy to reduce glucose levels should be a dynamic process tailored to each patient’s metabolic phenotype. Further research is crucial to understand the relationship between frailty, glycemic control, hypoglycemia, and metabolic phenotypes in older adults with diabetes. This includes defining optimal control targets and therapeutic strategies for each case [102].

Given the lack of consensus in defining frailty [49], it may be worth considering the development of a specific instrument tailored to individuals with diabetes [121]. Such a tool should encompass various factors beyond glycemic control (preferably with CGM variables associated with HbA_1c_), as well as hypoglycemia. This will account for not only the number and severity of hypoglycemic episodes but also falls [70], cognitive impairment, the duration of the disease [72], the burden of micro- and macrovascular complications, and the presence of chronic kidney disease and chronic liver disease. Other factors to consider are the presence of malnutrition, sarcopenia, obesity, MetS, and other markers of insulin resistance [122], such as triglyceride-glucose index [123] or visceral fat index with bioelectrical impedance analysis or abdominal ultrasound [14]. Gender-specific considerations should also be incorporated [124,125,126].

Addressing these knowledge gaps is essential for advancing scientific evidence and enhancing the management of older individuals living with diabetes and frailty.

## 5. Conclusions

The routine management of older adults with diabetes should include the detection of frailty, as it plays a significant role in their overall health. Further studies are needed to establish a relationship between frailty screening tools and factors such as glycemic control, hypoglycemia, and metabolic phenotypes. Among the tools reviewed, the Frailty Index, Physical Frailty Phenotype, and Clinical Frailty Scale are widely used and have been associated with key clinical outcomes in elderly individuals living with diabetes.

However, it is important to emphasize that this scoping review does not aim to recommend specific instruments over others. Instead, there is a pressing need to develop specific instruments tailored for this group of people considering their unique clinical-psychological-functional and social characteristics associated with geriatric syndromes.

By developing such precise tools, we can advance the field of precision medicine, allowing for tailored interventions that meet the individual needs of each older adult living with diabetes.

## Figures and Tables

**Table 1:** Clinicometric characteristics.

Frailty Instrument	Authors, Year	* Domains	Glycemic Control	Hypo Glycemia	Metabolic Phenotype
Clinical Frailty Scale-CSHA	Yanagita et al., 2018 [67]; Atif et al., 2019 [68]; MacKenzie et al., 2020 [69]	Ph, Ps	Yes	Yes	Yes
Continuous Frailty Scale	Wu et al., 2018 [62]	Ph	No	No	No
Edmonton Frail Scale-EFS	Carneiro et al., 2016 [70]; Aguayo et al., 2019 [71]; Bilgin et al., 2020 [72]; Akan et al., 2022 [73]	Ph, Ps, S	Yes	No	Yes
Electronic Frailty Index-eFI	Clegg et al., 2016 [64]	Ph	No	No	No
FRAIL scale	Liccini et al., 2016 [27]; Chao et al., 2018 [74]; Chao et al., 2020 [75]; Akan et al., 2022 [73]	Ph	No	Yes	Yes
Frailty Index (Rockwood’s FI)	Aguayo et al., 2019 [71]; Hyde et al., 2019 [76]; Ferri et al., 2020 [15]; Nguyen et al., 2021 [77]; Hanlon et al., 2021 [13]; Simpson et al., 2023 [78]; Saifuddin et al., 2023 [79]	Ph, Ps, S	Yes	Yes	Yes
Frailty Trait Scale-FTS	Garcia-Garcia et al., 2014 [55]; Castro et al., 2016 [80]	Ph	No	No	No
Physical Frailty Phenotype (Fried’s PFP)	Garcia-Esquinas et al., 2015 [81]; Zaslavsky et al., 2016 [82]; Kitamura et al., 2019 [83]; Aguayo et al., 2019 [71]; Hanlon et al., 2021 [13]; Saifuddin et al., 2023 [79]	Ph	Yes	Yes	Yes

* Domains: Ph: Physical; Ps: Psychological; S: Social.

## Data Availability

No new data were created or analyzed in this study. Data sharing is not applicable to this article as it is a review of existing literature.

## Abbreviations

CFS	Clinical Frailty Scale
CGM	Continuous Glucose Monitoring
COSMIN	Consensus-based Standards for the selection of Health Measurement Instruments
CSHA	Canadian Study of Health and Aging
eFI	Electronic Frailty Index
EFS	Edmonton Frail Scale
FI	Frailty Index
FTS	Frailty Trait Scale
HbA_1c_	Glycated hemoglobin
MetS	Metabolic Syndrome
PFP	Physical Frailty Phenotype
T2D	Type 2 diabetes
